# On Bedside Urine Testing: Including Quantitative Albumen and Sugar

**Published:** 1884-03

**Authors:** 


*On Bedside Urine Testing: including quantitative albumen and sugar.* Pp. 128. By GEO. OLIVER, M.D. Second edition. London: H. K. LEWIS. 1844.

This work is descriptive of what is essentially a new mode of urine testing, viz., by test papers. The reagents are deposited in a concentrated and readily soluble state in some chemically inert porous material such as filtering paper, and produce their effects by being re-dissolved in the urine. The most delicate tests were found to be potassic-mercuric-iodide, picric acid, and sodium tungstate. A sufficiently trustworthy and apparently easy method for the quantitative estimation of sugar and albumen is described.

Dr. Clifford's methods promise well, and should they prove trustworthy will no doubt be extensively used. His experiments have been well selected, and are carefully recorded; while the directions for the use of his tests are clear, concise and accurate. We heartily recommend the work to the perusal of our readers.

